# Integrated Transcriptome and Proteome Analysis Provides Insights into the Mechanism of *Blumea balsamifera* in Response to Drought Stress

**DOI:** 10.3390/biology15110861

**Published:** 2026-05-30

**Authors:** Zejun Mo, Changmao Guo, Su Chen, Kailang Mu, Shan Sha, Fei Ran, Pingxuan Xie, Changliu Shao, Zhigang Ju, Yuchen Liu, Yuan Yuan, Yuxin Pang

**Affiliations:** 1 College of Pharmaceutical Sciences, Guizhou University of Traditional Chinese Medicine, Guiyang 550025, China; zejmo66719@163.com (Z.M.); m18408568285@163.com (C.G.); cs920902@163.com (S.C.); mkl980818@163.com (K.M.); sha18932003204@163.com (S.S.); rf991125@163.com (F.R.); gdpuxpx@163.com (P.X.); shaochangliu178@163.com (C.S.); juzhigangz@163.com (Z.J.); liuyuchen480@gzy.edu.cn (Y.L.); 2 School of Biosciences and Biopharmaceutics, Guangdong Pharmaceutical University, Guangzhou 510006, China

**Keywords:** *Blumea balsamifera* L., drought stress, photosynthesis regulation, proteomics, roots, transcriptomics

## Abstract

Drought severely restricts the growth and secondary metabolism of medicinal plants. *Blumea balsamifera* is a water-sensitive and economically important medicinal species, yet its molecular regulatory mechanisms in response to drought remain largely unclear, which is worthy of in-depth investigation. In this study, four-month-old *B. balsamifera* seedlings were subjected to three treatments; normal irrigation (CK), drought stress (DS), and rehydration recovery (RW). Leaf photosynthetic parameters, L-Borneol content, and root physiological indices were determined, and transcriptomic and proteomic analyses were integrated to explore its drought response mechanism. Under drought stress, leaf net photosynthetic rate, transpiration rate, and stomatal conductance decreased sharply, while intercellular CO_2_ concentration increased; L-Borneol content showed a biphasic change, and root malondialdehyde content accumulated continuously, accompanied by significant increases in antioxidant enzyme activities and osmotic regulator contents. A total of 9917 differentially expressed genes and 736 differentially expressed proteins were identified, which were mainly enriched in phenylpropanoid biosynthesis, photosynthesis and other pathways, with photosynthesis-related genes and proteins coordinately downregulated. *B. balsamifera* adapts to drought stress by activating the antioxidant defense system, regulating osmotic substances, and reprogramming photosynthetic networks. The key candidate genes obtained provide important targets for drought-tolerant breeding of this species, and their reliability was verified by RT-qPCR.

## Simple Summary

Drought limits the growth and secondary metabolism of medicinal *Blumea balsamifera*, and its molecular mechanism of drought response remains unclear. We integrated physiological, transcriptomic and proteomic analyses to explore its drought adaptation. Drought repressed photosynthesis, altered L-Borneol accumulation, activated root antioxidant and osmotic systems, and downregulated photosynthesis-related genes and proteins enriched in phenylpropanoid biosynthesis. This study clarifies the drought-tolerance mechanism of *B. balsamifera* and provides candidate genes for its drought-resistant breeding.

## 1. Introduction

Drought, as one of the major environmental challenges faced by global agricultural systems, severely impacts plant growth and development, secondary metabolite accumulation, and medicinal value [1,2,3]. Due to their complex metabolic pathways and diverse stress resistance mechanisms, studies on the stress responses of medicinal plants are crucial for the sustainable utilization of resources [4,5]. As the primary organ for perceiving and responding to water stress, plant roots play irreplaceable roles in maintaining water absorption efficiency and stress signal transduction through mechanisms such as cell wall remodeling [6], physiological adaptation [7], and microbial interactions [8].

Photosynthesis, as the core of plant energy metabolism, is predominantly impacted by drought stress via structural and functional alterations to the photosynthetic apparatus, thereby reducing photosynthetic efficiency and subsequently affecting plant growth [9]. Stomatal limitation primarily drives photosynthetic rate (Pn) decline, manifested as reduced intercellular CO_2_ concentration (Ci) resulting from decreased stomatal conductance (Gs) [10]. Under prolonged stress, however, damage to thylakoid membranes and photosynthetic enzymes renders non-stomatal limitation dominant [11]. Drought stress also induces excessive reactive oxygen species (ROS) accumulation, triggering membrane lipid peroxidation and cellular damage [12]. Plants activate antioxidant defense systems involving SOD, CAT and POD to scavenge reactive oxygen species and alleviate oxidative stress damage [13]. Concurrently, osmolyte accumulation (e.g., proline, soluble sugars) maintains cellular osmotic homeostasis under water deficit conditions [14,15].

Integrated transcriptomics–proteomics analyses have emerged as a robust methodology to dissect the molecular mechanisms underlying plant drought responses, particularly for elucidating drought stress regulatory networks in secondary metabolite biosynthesis of medicinal plants [16]. For instance, Feng et al. [17] identified phenylpropanoid biosynthesis-associated genes/proteins and plant hormone signaling pathways as key components in *Arabidopsis thaliana* drought tolerance regulation. Kong et al. [18] revealed abscisic acid (ABA)-mediated regulatory cascades in *Panax ginseng* under water deficit conditions. Wang et al. [19] demonstrated that *Chrysanthemum CmNF-YB8* gene compromises drought resistance by downregulating stomatal regulatory gene *CmCIPK6* and cuticle synthesis gene *CmSHN3*, resulting in enlarged stomatal aperture and reduced cuticle thickness. In *Salvia miltiorrhiza*, drought stress enhances tanshinone (terpenoid secondary metabolite) accumulation via activating overexpression of the terpenoid synthase gene *SmAHL* [20].

*Blumea balsamifera* L.DC., a perennial medicinal herb, serves as a primary botanical source of traditional Chinese medicine “Borneol” [21]. Its leaves are rich in terpenoids (e.g., L-Borneol), which exhibit antibacterial, antitumor, and analgesic pharmacological activities [22]. However, *B. balsamifera* frequently encounters seasonal drought stress in its natural habitat [23]. Drought stress not only impairs the plant’s photosynthetic efficiency [24] and biomass accumulation [25] but also potentially alters the biosynthesis and allocation of bioactive compounds by regulating secondary metabolic pathways [26]. As the primary organ for drought signal perception, the root system likely plays a pivotal role in *B. balsamifera*’s drought stress response. Nevertheless, the molecular mechanisms underlying root–leaf signal transduction under a water deficit remain poorly understood.

Against this backdrop, this study employed *B. balsamifera* seedlings as plant materials. Through the application of three experimental treatments—normal irrigation (CK), continuous drought stress (DS), and rehydration recovery (RW)—combined with physiological–biochemical parameter measurements and transcriptome–proteome integrated analysis, we systematically investigated the impacts of drought stress on root antioxidant systems, osmotic adjustment capacity, and photosynthetic metabolism in *B. balsamifera.* The objective is to provide a theoretical basis for clarifying the molecular signal transduction mechanisms of drought response in *B. balsamifera* and screening key drought-tolerant candidate genes.

## 2. Materials and Methods

### 2.1. Test Materials and Drought

Seeds of *B. balsamifera* were sourced from Hainan Tropical Botanical Garden (19°30′36″ N, 109°34′12″ E) and taxonomically identified as Asteraceae species *B. balsamifera* by Professor Pang Yuxin of Guizhou University of Traditional Chinese Medicine following the classification standards of Flora Reipublicae Popularis Sinicae. All procedures pertaining to plant material collection and experimental implementation were performed in accordance with institutional, national and international regulatory guidelines. The obtained seeds were subsequently germinated and cultivated in the No.2 greenhouse (26°22′20″ N, 106°27′34″ E) at the Medicinal Plant Cultivation Experimental Base of Guizhou University of Traditional Chinese Medicine.

In August 2024, four-month-old *B. balsamifera* seedlings with consistent growth vigor and an average plant height of 18.76 cm were selected for the experiment. Three experimental groups were established: well-watered control (CK), drought stress treatment (DS), and rehydration recovery group (RW). The experimental regime was formulated based on published drought stress protocols [27] and our preliminary experimental data. Pre-experimental results confirmed that *B. balsamifera* seedlings suffered irreversible mortality under continuous drought for 20 days and could not recover after rewatering. To avoid complete seedling death in the DS group and guarantee the effective recovery performance as well as rehydration compensation effects of the RW group, the 16-day drought treatment cycle was determined through comprehensive evaluation. The CK group received daily full irrigation to pot bottom water seepage every evening. The DS group was subjected to continuous drought after a single irrigation. The RW group was rewatered to pot bottom seepage on the 8th, 10th, 12th, and 14th day of drought stress. Sampling time points were set at 0, 5, 8, 10, 12, 14, and 16 days, with the RW group sampled 2 days after rewatering. In each treatment group, 3 seedlings were randomly selected at each sampling time. Whole roots were harvested: rhizospheric soil debris was removed, roots were washed with sterile water and air-dried, then excised from the base with sterile scissors. After cutting into pieces and homogenizing, root samples were divided into sterile cryotubes, immediately frozen in liquid nitrogen, and stored at −80 °C for subsequent analysis.

### 2.2. Determination of Photosynthetic Parameters in Leaves

On day 16 of the treatment, between 09:00 and 11:00 h, a LCI portable photosynthesis system (Beijing Aozuo Ecological Instrument Co., Ltd., 19B, Hengxing Building, No.89 Zhongguancun East Road, Haidian District, Beijing, China) was used to measure net photosynthetic rate (Pn), transpiration rate (Tr), intercellular CO_2_ concentration (Ci), and stomatal conductance (Gs) in *B. balsamifera* seedlings at a constant light intensity of 800 μmol·m^−2^·s^−1^.

### 2.3. Determination of L-Borneol Content in Leaves

The L-Borneol content in *B. balsamifera* leaves was quantified using gas chromatography (GC) following a validated method [28]. Analysis was performed on a Shimadzu GC-2010 Pro system equipped with a flame ionization detector (FID) and AOC-20i auto-injector (Shimadzu Corporation, Kyoto, Japan), using an HP-5 capillary column (30 m × 0.25 mm × 0.25 μm; Agilent Technologies, Santa Clara, CA, USA). Instrument parameters were set as follows: injector and detector temperatures maintained at 240 °C, nitrogen carrier gas flow rate of 2.0 mL/min, and split injection mode (split ratio 20:1) with 1 μL injection volume. The oven temperature program began at 90 °C (hold 2 min), increased to 100 °C at 4 °C/min, then ramped to 160 °C at 20 °C/min (hold 6 min). Method validation included assessments of linearity, precision, stability, and recovery.

### 2.4. Determination of Physiological and Biochemical Indices in Roots

Antioxidant system responses in *B. balsamifera* seedling roots were evaluated by quantifying malondialdehyde (MDA) content via the thiobarbituric acid (TBA) assay [29], superoxide dismutase (SOD) activity using the WST-8 method [30], catalase (CAT) activity by ultraviolet spectrophotometry [31], and peroxidase (POD) activity through the guaiacol peroxidase assay [32]. Concurrently, osmotic adjustment substance responses were investigated by measuring root lignin (LIG) content using the acetylation method [33], soluble sugar (SS) content via anthrone colorimetry [34], soluble protein (SP) content by the Coomassie Brilliant Blue assay [35], and proline (PRO) content through the ninhydrin colorimetric method [36].

### 2.5. Selection of Sequencing Time Points and Samples

Comprehensive physiological and biochemical assays on the roots of *B. balsamifera* revealed significant variations in parameters on the 10th day of drought stress, suggesting that the plants had entered a critical transition stage from stress acclimation to drought response. Given the hypothesis that differential gene expression drives this transition, transcriptome sequencing was performed at this time point to identify key differentially expressed genes (DEGs) and their underlying regulatory mechanisms.

### 2.6. Transcriptome Analysis

On day 10 of the treatment, three biological replicates of root samples from CK (control) and DS (drought stress) groups were selected for transcriptome sequencing by a Total RNA Extraction Kit (Shanghai Meiji Biomedical Technology Co., Ltd., Shanghai, China). A Nanodrop 2000 spectrophotometer (Thermo Fisher Scientific, Waltham, MA, USA) was applied to detect RNA concentration and purity, while agarose gel electrophoresis and an Agilent 5300 bioanalyzer (Agilent Technologies, USA) were separately used to assess RNA integrity and determine the RNA integrity number (RIN). After preliminary quality inspection, raw paired-end reads were processed and filtered with the fastp program (https://github.com/OpenGene/fastp (accessed on 6 December 2025)) [37]. Reads contaminated with adapter sequences, those containing over 5% ambiguous bases (N), and sequences with more than 20% bases scoring ≤20 in quality were eliminated. The retained high-quality clean reads were adopted for further bioinformatic analysis.

De novo assembly of all clean reads was initially performed using Trinity software (https://github.com/trinityrnaseq/trinityrnaseq/wiki (accessed on 6 December 2025)) to generate primary Unigenes [38]. Initial Unigene sequences were then clustered using the CD-HIT software (http://weizhongli-lab.org/cd-hit/ (accessed on 6 December 2025)) to remove redundancy, with a sequence identity threshold set at 0.95. Additionally, sequences shorter than 200 bp were filtered to obtain non-redundant Unigenes. Differential gene expression analysis was performed using DESeq2 software (http://bioconductor.org/packages/stats/bioc/DESeq2/ (accessed on 6 December 2025)) [39], with thresholds set at |log_2_FC| ≥ 1 and an adjusted *p*-value. Genes were clustered by expression patterns across samples, and differentially expressed genes (DEGs) were functionally annotated and enriched against the Gene Ontology (GO; http://www.geneontology.org/ (accessed on 6 December 2025)) and Kyoto Encyclopedia of Genes and Genomes (KEGG; http://www.genome.jp/kegg/ (accessed on 6 December 2025)) databases.

### 2.7. Proteome Analyses

On day 10 of treatment, three biological replicates of CK and DS root samples were ground to a fine powder using liquid nitrogen. Protein extraction was performed using a lysis buffer (8 M urea + 1% SDS containing protease inhibitor cocktail), and the resulting protein supernatant was subdivided for downstream analyses: one portion for online analysis and the other for SDS-PAGE electrophoresis.

TMT labeling and mass spectrometry were conducted by Shanghai Meiji Biotechnology Co., Ltd. (Shanghai, China). Samples underwent one-dimensional separation via reverse-phase liquid chromatography (RPLC), followed by two-dimensional separation using nano-liquid chromatography tandem mass spectrometry (EasynLC 1200 system coupled with a Q Exactive mass spectrometer, Thermo Fisher Scientific Inc., Waltham, MA, USA). Mass spectrometry-derived protein sequences were annotated by aligning against the EggNOG, GO, KEGG, NR, Pfam, String, and Uniprot databases. Differential protein analysis was performed based on quantitative results, with differentially expressed proteins (DEPs) identified using field-standard criteria: |log_2_FC| ≥ 1 and an adjusted *p*-value (FDR correction) < 0.05 [40].

### 2.8. Association Analysis Between Transcriptome and Proteome

We engaged Shanghai Meiji Biotechnology Co., Ltd. to perform multi-omics correlation analysis of transcriptome and proteome sequencing data under drought stress in *B. balsamifera*, aiming to investigate molecular expression characteristics at both RNA and protein levels and decipher key molecular mechanisms of drought response. Transcriptional DEGs and post-translational DEPs were paired as DEG-DEP pairs. Differential mRNA–protein correlations across sample groups were visualized using Venn diagrams and cluster heatmaps, with quantitative analysis performed via Pearson correlation coefficients.

Functional annotation and enrichment analysis of DEG-DEP pairs were conducted using the blastp function of Diamond software (https://github.com/bbuchfink/diamond (accessed on 6 December 2025)) against GO and KEGG databases for in-depth data mining. Drought-associated protein–protein interaction (PPI) networks were constructed using the STRING database (https://string-db.org/ (accessed on 6 December 2025)), and gene co-regulation networks were visualized via Cytoscape software (https://cytoscape.org/ (accessed on 6 December 2025)).

### 2.9. Determination of Gene Expression Levels (RT-qPCR)

To validate the reliability of mRNA differential expression detected by high-throughput sequencing, 12 transcripts were selected from DEGs/DEPs with associations identified in integrated transcriptome–proteome analysis for RT-qPCR verification, and the RT-qPCR results were subsequently compared with those of RNA-Seq analysis. Total RNA was isolated using the MJZol Total RNA Extraction Kit (Shanghai Meiji Biomedical Technology Co., Ltd.). Qualified RNA was reverse-transcribed into cDNA with ExonScript RTMix (containing dsDNase), using 18S rRNA as the internal reference gene (forward primer: CGGCTACCACATCCAAGGAA; reverse primer: GCTGGAATTACCGCGGCT). Experimental procedures were performed in accordance with the instructions of the SYBR Prime qPCR Kit (FastHS, product code BG0014). For RT-qPCR, three biological replicates (each from independent plants in the same treatment group) were set for each time point, with three technical replicates per biological replicate. The genes and corresponding primers used in qPCR are listed in Table S1, and the relative expression level of target genes was calculated via the CT method (2^−△△Ct^).

### 2.10. Data Statistics and Analysis

SPSS 22.0 and Excel 2021 were employed for statistical analysis of experimental data, while GraphPad Prism 9.5 and OriginPro 2024 were used for graph construction. All data were expressed as the mean ± standard deviation (mean ± SD) of three replicates. Duncan’s new multiple range test was applied to determine significant differences among groups, where different lowercase letters denoted significant differences between different treatments at the same drought time point (*p* < 0.05).

## 3. Results

### 3.1. Changes in Photosynthetic Characteristics and L-Borneol Content in Leaves

**Figure 1:**
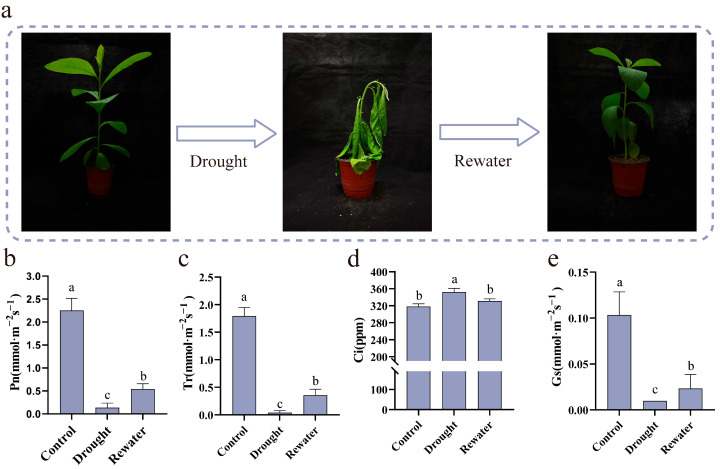
Effects of drought stress on growth morphology and photosynthetic parameters of *B. balsamifera*. (**a**) Phenotypic characters. (**b**–**e**) Pn, Tr, Ci and Gs. Different lowercase letters in the graphs indicate significant differences (*p* < 0.05), and same lowercase letters indicate no significant differences (*p* > 0.05).

*B. balsamifera* exhibited obvious morphological changes under drought stress, with observed wilting. After rehydration, the plants resumed a relatively normal growth state (Figure 1a). Regarding photosynthetic parameters, compared with CK, drought stress significantly decreased Pn, Tr, and Gs of *B. balsamifera* (Figure 1b,c,e), with respective reductions of 93.78%, 99.71%, and 90.34%. However, Ci increased by 10.68% under drought stress, which may be associated with damage to leaf photosynthetic structures (Figure 1d). Following rehydration, Pn, Tr, and Gs increased significantly but did not fully recover to control levels, indicating that drought stress impacts on the photosynthetic system of *B. balsamifera* were not fully reversible via rehydration.

Table 1 presents L-Borneol mass fractions in *B. balsamifera* leaves under drought stress and rewatering at different treatment times. There was no significant difference in the mass fraction of L-Borneol between CK and DS from 0 to 5 days of treatment. After 8 days of drought stress, DS exhibited a significantly higher mass fraction than CK, peaking at (0.969 ± 0.005) mg·g^−1^ after 12 days of treatment. At day 8 of rewatering (RW), the mass fraction was (0.166 ± 0.013) mg·g^−1^—significantly lower than DS but higher than CK. Combined with the typical temporal characteristics of root signal transduction under drought stress, significant L-borneol accumulation in leaves from 8 days post-stress suggests a temporal correlation between its accumulation dynamics and root signal response processes following stress perception.

### 3.2. Physiological and Biochemical Responses of Roots

**Figure 2:**
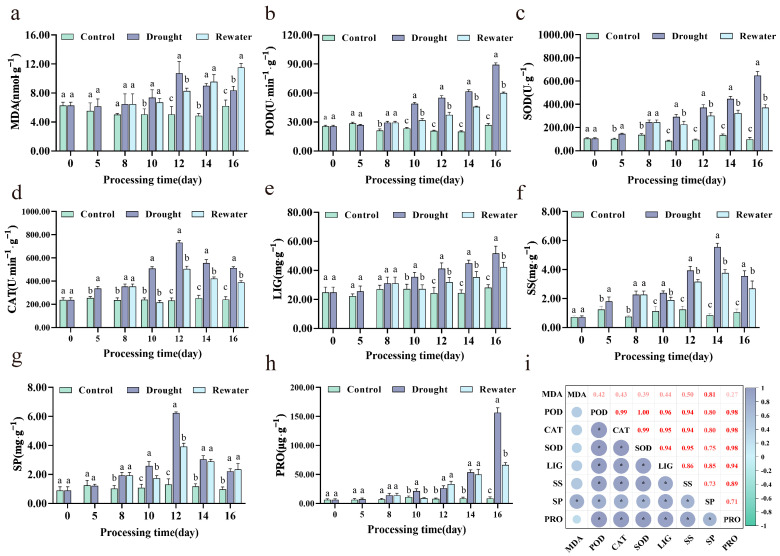
Effect of drought stress on root physiology and biochemistry of *B. balsamifera.* (**a**–**d**) MDA content, POD, SOD and CAT activities. (**e**–**h**) LIG, SS, SP and PRO contents. (**i**) Correlation analysis of physiological and biochemical indexes, purple represents positive correlation, green represents negative correlation, and the numbers indicate correlation coefficients. Different lowercase letters in the graphs indicate significant differences (*p* < 0.05), and the same lowercase letters indicate no significant differences (*p* > 0.05). *: *p* < 0.05.

During DS treatment, MDA content in *B. balsamifera* roots exhibited a continuous increase, reaching its peak on day 12, which indicated that drought stress progressively enhanced membrane lipid peroxidation (Figure 2a). In the antioxidant enzyme system, POD and SOD activities were significantly enhanced with prolonged stress duration, peaking at treatment termination. CAT activity initially increased and then decreased (Figure 2b–d). Additionally, drought stress significantly elevated LIG content (Figure 2e). SS and SP levels peaked on day 14 and day 12, respectively, before declining, both significantly higher than CK. PRO content continuously rose, reaching 156.50 μg·g^−1^ at treatment end—17.99-fold higher than CK and significantly exceeding RW levels (Figure 2f–h).

After re-watering, MDA content, antioxidant enzyme activities, and osmolyte levels in *B. balsamifera* roots were significantly downregulated compared to DS, suggesting the plant activated physiological repair mechanisms to mitigate drought-induced damage. However, due to stress duration limitations, physiological indices could not fully recover to baseline levels. Correlation analysis (Figure 2i) revealed positive correlations between MDA and antioxidant enzyme activities, indicating a close association between membrane lipid peroxidation severity and antioxidant defense system activation. Osmolytes were correlated with antioxidant enzyme activities, demonstrating that *B. balsamifera* maintained intracellular homeostasis via synergistic regulation across multiple indices during drought and rehydration.

### 3.3. Transcriptome Sequencing Analysis

**Figure 3:**
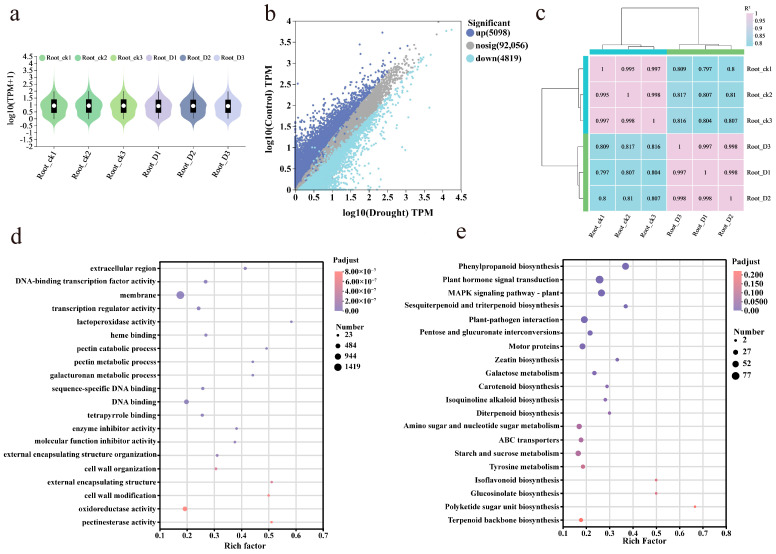
Transcriptome expression analysis. (**a**) Violin diagram. (**b**) Gene volcano map. In the figure, blue dots represent downregulated differentially expressed genes, purple dots represent upregulated genes, and gray dots represent non-significant differentially expressed genes. (**c**) Sample correlation heat map. (**d**) GO functional enrichment of DEGs. (**e**) KEGG functional enrichment of DEGs.

Quality control statistics of sequencing data are presented in Supplementary Materials, Table S2, while comparisons between sequencing and assembly results are shown in Supplementary Materials, Table S3. Root tissue samples of *B. balsamifera* exhibited relatively high overall transcriptional expression levels. Notable variations in gene expression level distributions were observed between the CK and DS groups, whereas gene expression patterns within the same group showed high consistency (Figure 3a–c). Compared with the CK group, a total of 9917 DEGs were identified in the DS group, including 5098 upregulated and 4819 downregulated genes (Figure 3c). DEGs were significantly enriched in GO categories such as extracellular region, DNA-binding transcription factor activity, and membrane (Figure 3d). KEGG analysis revealed significant enrichment in pathways including phenylpropanoid biosynthesis, plant hormone signal transduction, and plant MAPK signaling, indicating these pathways are crucial for *B. balsamifera’s* response to drought stress and may potentially participate in regulating plant physiological and biochemical mechanisms (Figure 3e).

### 3.4. Proteome Sequencing Analysis

**Figure 4:**
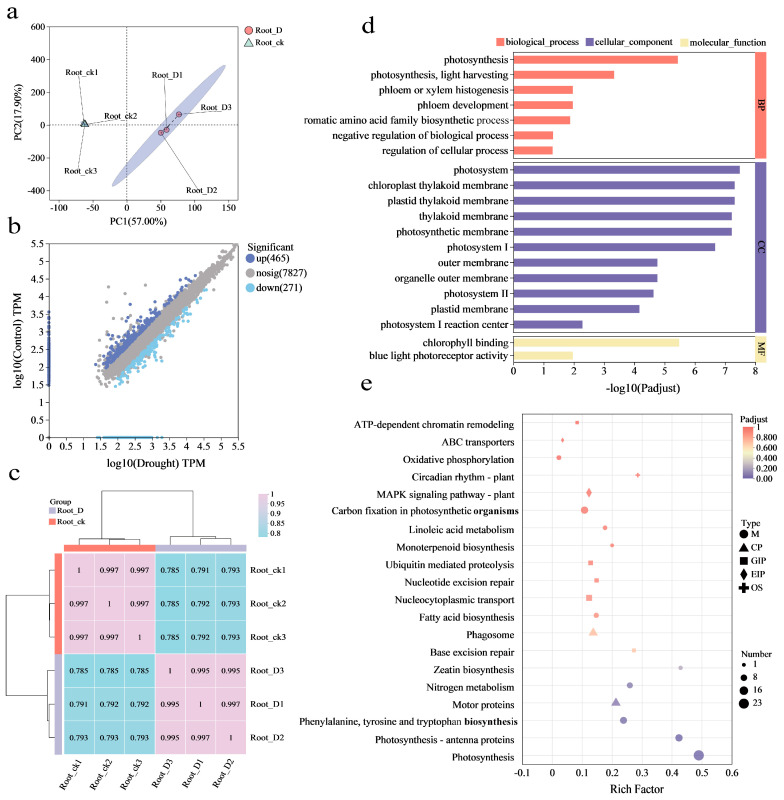
Proteome expression analysis. (**a**) Principal component analysis. Each point represents a repetition in the grouping experiment, and different colors distinguish different groups. (**b**) Volcano map of identified genes. In the figure, blue dots represent downregulated differentially expressed genes, red dots represent upregulated genes, and gray dots represent non-significant differentially expressed genes. (**c**) Sample correlation heat map. (**d**) GO functional enrichment of DEGs. (**e**) KEGG functional enrichment of DEGs.

Principal component analysis and cluster analysis (Figure 4a,c) revealed tight clustering of samples within groups, demonstrating high reproducibility, and distinct separation between groups, reflecting the impact of drought stress on proteome expression. Based on proteome data, 736 DEPs were identified, including 465 upregulated and 271 downregulated proteins (Figure 4b). GO enrichment analysis showed significant enrichment of DEPs in biological processes such as photosynthesis and light harvesting. In cellular component categories, the photosystem and chloroplast thylakoid membrane were significantly enriched. For molecular functions, chlorophyll binding and blue light photoreceptor activity terms were highly enriched (Figure 4d). KEGG enrichment analysis identified significant enrichment of DEPs in pathways including ATP-dependent chromatin remodeling, ABC transporters, oxidative phosphorylation, circadian rhythm–plant, and the plant MAPK signaling pathway (Figure 4e).

Collectively, these findings indicate that *B*. *balsamifera* under drought stress reorganizes cellular physiological processes to adapt to drought by regulating photosynthesis-associated cellular structures, molecular functions, and core protein expression across multiple pathways, including energy metabolism, substance transport, and signal transduction.

### 3.5. Correlation Analysis and Functional Enrichment of DEGs-DEPs

**Figure 5:**
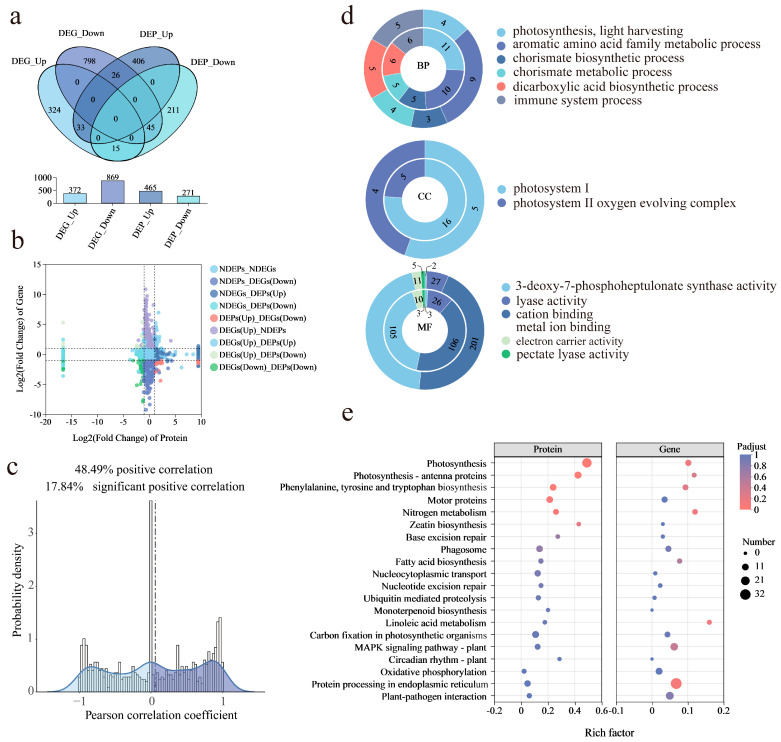
Combined analysis of transcriptome and proteome. (**a**) Comparative analysis of DEGs-DEPs in CK group and DS group. (**b**) Nine-quadrant analysis of mRNA and protein. (**c**) Correlation analysis between mRNA and protein. The abscissa is the numerical range of the correlation coefficient, and the ordinate is the density distribution of the correlation coefficient. (**d**) GO enrichment analysis of DEGs-DEPs. (**e**) KEGG pathway analysis of DEGs-DEPs.

Integrated proteomics and transcriptomics analyses were performed to assess the correlation between the two omics datasets. Transcriptomic profiling identified 1241 differentially expressed genes (DEGs), while proteomic analysis detected 736 differentially expressed proteins (DEPs). A total of 119 common genes were co-identified in both datasets (Figure 5a,b). In the comparison of the CK group versus the DS group, proteomics revealed 465 upregulated and 271 downregulated DEPs, while transcriptomics identified 372 upregulated and 869 downregulated DEGs. Among the common genes, 45 DEPs and their corresponding DEGs were co-downregulated, and 33 were co-upregulated (co-regulated DEG-DEP pairs). Conversely, 15 DEPs were downregulated while their DEGs were upregulated, and 26 DEPs were upregulated while their DEGs were downregulated (inversely regulated pairs). Pearson correlation analysis evaluated the proteome–transcriptome correlation, showing moderate overall consistency between mRNA and protein expression. Specifically, 48.49% of pairs exhibited positive correlation, with 17.84% showing significant positive correlation (Figure 5c).

GO enrichment analysis revealed that DEPs were significantly enriched in biological processes including photosynthesis, light harvesting, and aromatic amino acid metabolic processes. In the cellular component category, enrichment was observed in Photosystem I, Photosystem II oxygen-evolving complex, and other chloroplast-related structures. For molecular functions, DEPs were predominantly enriched in 3-deoxy-7-phosphoheptulonate synthase activity, lyase activity, and other catalytic functions (Figure 5d). KEGG pathway analysis of co-identified DEGs and DEPs showed significant enrichment in photosynthesis, photosynthesis–antenna proteins, and phenylalanine/tyrosine/tryptophan biosynthesis pathways. Collectively, these results suggest that *B. balsamifera* may exhibit adaptive responses to drought stress by modulating photosynthetic machinery, aromatic amino acid metabolism, and associated key enzymes and components (Figure 5e).

### 3.6. DEPs and DEGs Associated with Drought Tolerance and PPI Network Analysis

**Figure 6:**
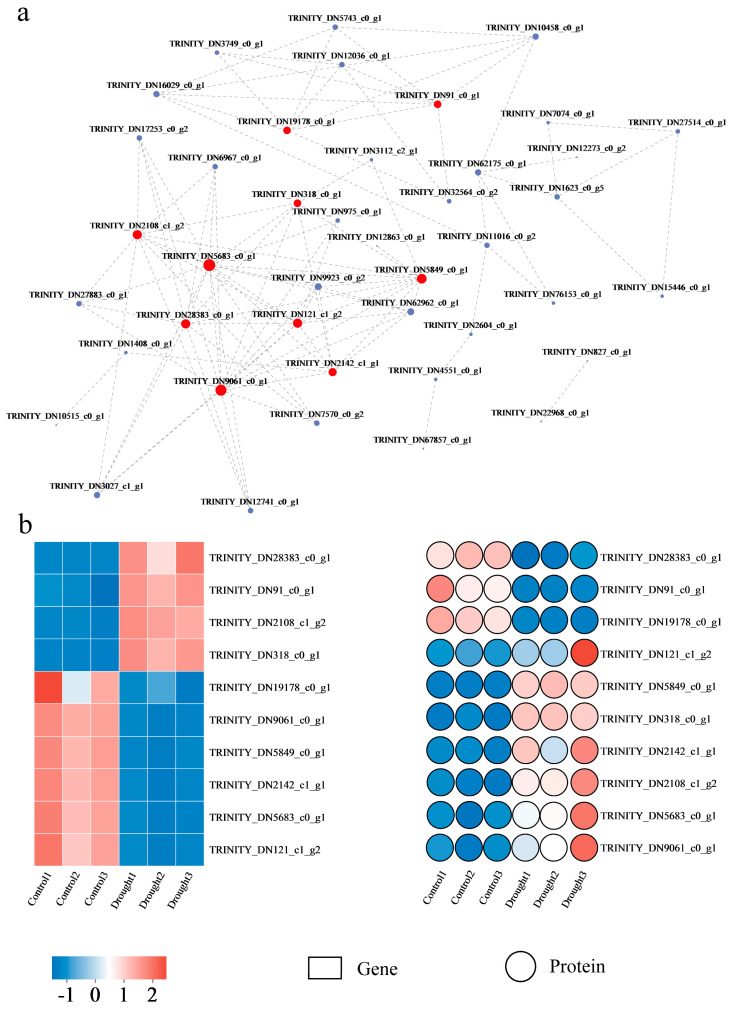
Combined analysis of transcriptome and proteome. (**a**) Co-expression network of DEGs-DEPs related to drought tolerance. The size of the circle represents the number of interactions with genes or proteins, and the red dots represent the top 10 key genes. (**b**) The expression of the first 10 DEGs-DEPs related to drought tolerance, the rectangle represents the gene and the circle represents the protein.

To screen key drought-tolerant genes in *B. balsamifera* roots, 63 co-identified DEGs and DEPs were selected from five drought-related pathways: Photosynthesis, Protein Processing in Endoplasmic Reticulum, Phagosome, Carbon Fixation in Photosynthetic Organisms, and Plant–Pathogen Interaction. A protein–protein interaction (PPI) network was constructed using the STRING database (https://string-db.org (accessed on 6 December 2025)) and visualized via Cytoscape software (https://cytoscape.org (accessed on 6 December 2025)), comprising 30 nodes (Figure 6a). Notably, TRINITY_DN5683_c0_g1 (Trinity transcript ID), TRINITY_DN9061_c0_g1 (Trinity transcript ID), and TRINITY_DN5849_c0_g1 (Trinity transcript ID) exhibited ≥10 interaction edges, suggesting their roles as hub nodes in the drought-resistance regulatory network of *B. balsamifera* (Table S4).

Hierarchical cluster analysis revealed that the top 10 co-identified DEGs and DEPs associated with drought tolerance exhibited divergent expression patterns at the gene and protein levels under drought stress: the expression of six node genes showed significant downregulation, whereas their corresponding proteins were significantly upregulated. This expression pattern discrepancy was primarily attributed to post-transcriptional regulation, protein stability control, and cellular adaptive strategies under drought stress. Notably, TRINITY_DN2108_c1_g2 (Trinity transcript ID) and TRINITY_DN318_c0_g1 (Trinity transcript ID) and their corresponding proteins were consistently upregulated in *B. balsamifera* under drought stress, suggesting these genes may play pivotal roles in drought tolerance by coordinating upregulation at both transcriptional and translational levels.

### 3.7. Regulation of DEGs and DEPs in Photosynthesis

**Figure 7:**
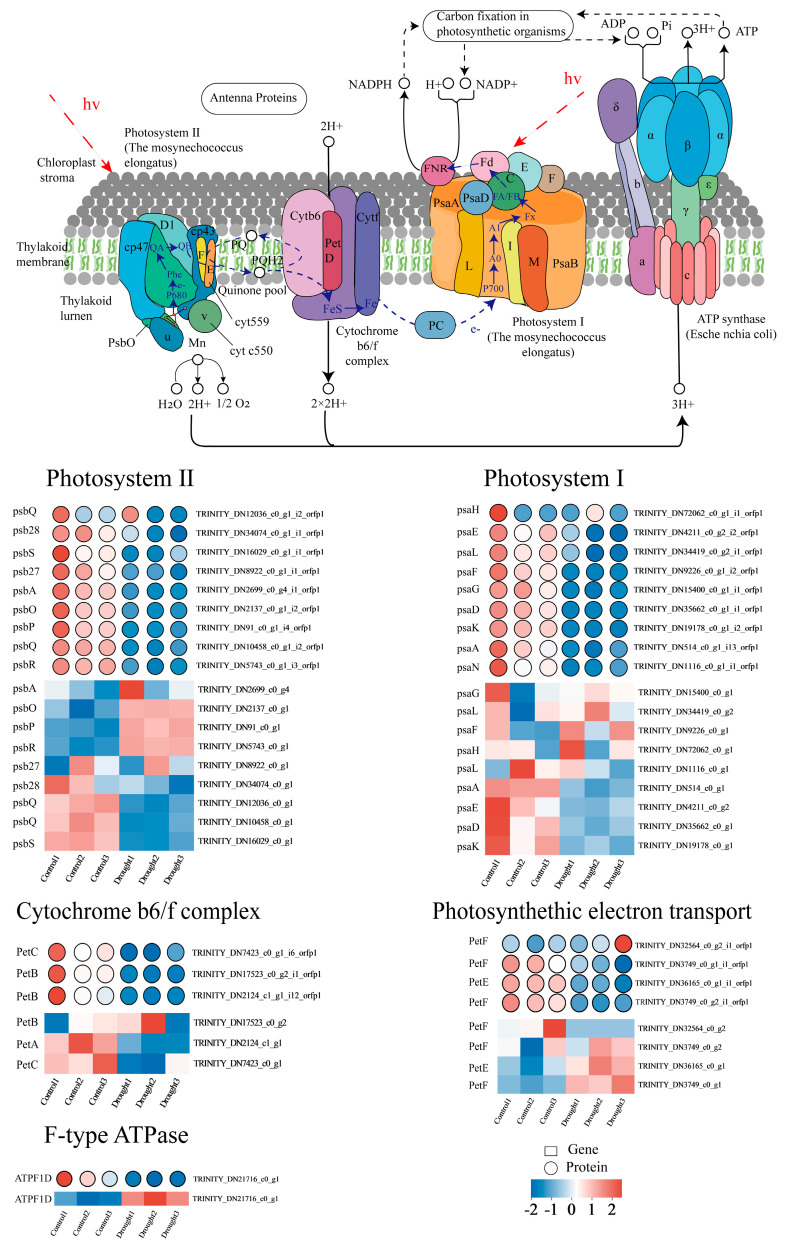
Differences in the expression of photosynthetic metabolic pathways between transcriptome and proteome. Blue indicates low expression and red indicates high expression. The high-abundance metabolites are expressed in deep red, and the low-abundance metabolites are expressed in deep blue. **Left**: *PSbA-PSb28* is Photosystem II-related protein coding gene; *psaA-psaN* is the key gene encoding the protein of Photosystem I; *petA*, *petB* and *petC* are the coding genes of Cytochrome b6/f complex. *petE* and *petF* are photosynthetic electron transport-related protein-coding genes. *ATPF1D* is an F-type ATPase-related protein-coding gene. **Right**: Gene or protein number.

Drought stress significantly impacts plant photosynthetic metabolic pathways. To dissect the molecular response mechanisms of *B. balsamifera* under drought stress, we analyzed transcriptomic and proteomic expression profiles within photosynthetic pathways (Figure 7). Results showed that transcripts and proteins of key photosynthetic components exhibited coordinated expression patterns, including *PsbA*, *Psb28*, *PsbQ*, and *PsbS* in Photosystem II; *PsaL*, *PsaA*, *PsaE*, *PsaD*, and *PsaK* in Photosystem I; *PetA* and *PetB* in the cytochrome b6/f complex; and photosynthetic electron transport-related genes (e.g., *PetE*).

However, transcription levels of photosynthetic pathway genes were not always congruent with protein abundances, a discrepancy suggested to involve post-transcriptional and post-translational regulatory mechanisms. Notably, coordinately downregulated DEG-DEP pairs (e.g., *PsbQ*, *Psb28*, *PsaK*) indicated drought-induced functional inhibition of their encoded proteins, potentially impairing overall photosystem activity. Expression changes in cytochrome b6/f complex components (*PetA*, *PetB*) may disrupt electron transport chain activity, thereby reducing photosynthetic electron transport efficiency. Alterations in photosynthetic electron transport genes (*PetE*) reflected drought impacts on this pathway, while differential expression of F-type ATPase-related genes (*ATPF1D*) could directly compromise cellular energy supply.

### 3.8. Validation of RNA-Seq Data

**Figure 8:**
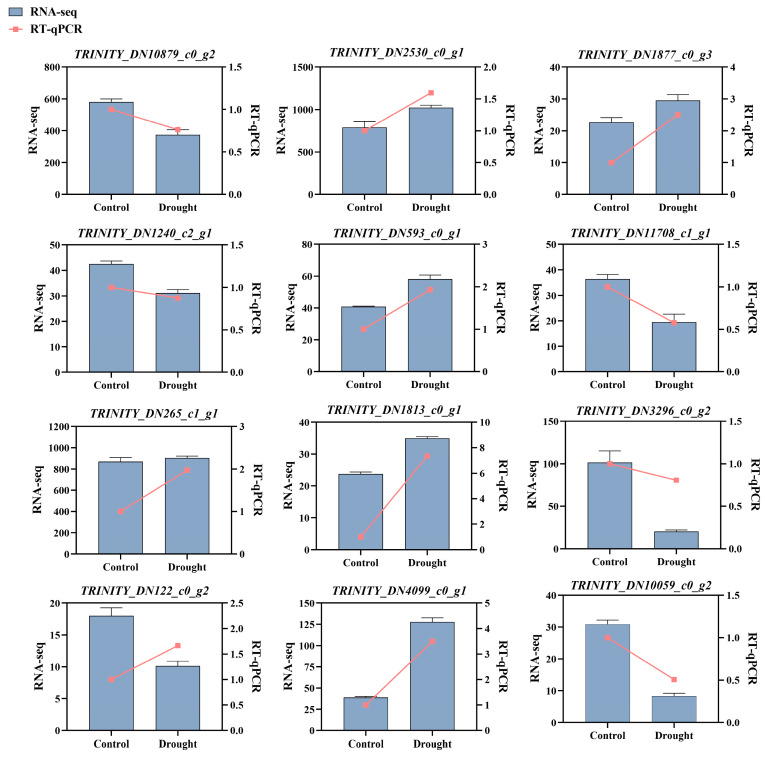
RT-qPCR comparative analysis.

To validate the reliability of RNA-seq results, 12 genes were selected for RT-qPCR analysis of their expression patterns under CK and DS treatments (Figure 8, Table S1). Results showed that 11 genes exhibited concordant expression profiles between RNA-seq and RT-qPCR analyses (Figure 8). These findings demonstrate high consistency between RNA-seq data and RT-qPCR validation results, providing a robust foundation for subsequent functional gene mining.

## 4. Discussion

Drought stress, a major environmental factor influencing plant growth and development, impacts plant adaptability by disrupting photosynthesis, impairing cellular homeostasis, and regulating gene expression [41,42]. In this study, drought stress significantly reduced the Pn, Tr and Gs in *B. balsamifera* leaves, consistent with findings in *Arabidopsis thaliana* [43], *Cinnamomum camphora* [44], and *Cyclocodon lancifolius* [45]. Notably, Ci increased significantly post-drought treatment, accompanied by coordinated downregulation of photosynthesis-related DEG-DEP pairs, including PSII core subunit gene *psbA*, PSI subunit gene *psaK*, and cytochrome b6/f complex subunit genes *PetA/PetB*. These DEG-DEP pairs were not randomly downregulated but showed tight co-expression patterns, and PPI network analysis revealed that several of these photosynthesis-related DEPs (e.g., *PsbA*, *PsaK*) interact with the core hub genes (*SHD* and *CRT*) identified in our integrated analysis. This interaction suggests that the core hub genes may directly regulate the transcription and translation of these photosynthetic genes, thereby mediating the downregulation of photosynthetic apparatus components. These results indicate that non-stomatal limitations (damage to the photosynthetic apparatus) are the primary drivers of reduced photosynthetic efficiency [46]. Structural impairment of photosynthetic complexes likely underlies the controlled downregulation of the linear electron transport pathway. Future studies will supplement multi-time-point physiological detection across progressive drought gradients to compensate for this limitation and further elaborate the staged response characteristics of photosynthesis in *B. balsamifera* under drought stress.

Drought stress significantly induced L-borneol accumulation in *B. balsamifera* leaves, which corresponded to differential expression of terpenoid biosynthesis pathway genes in the transcriptome (Table S5). With the prolongation of drought treatment, persistent water deficit signals gradually activated the transcription of key genes in the terpenoid biosynthesis pathway, thereby driving the continuous enrichment of L-borneol in leaf tissues. This time-dependent elevation of L-borneol content serves as a passive adaptive metabolic response of *B. balsamifera* to progressive drought stress. Terpenoid metabolites are known to maintain cell membrane integrity by regulating membrane lipid composition; for instance, drought-induced accumulation of the ABA enhances plant cell antioxidant capacity [47]. Combined with L-borneol’s role as a membrane lipid component regulator, we speculate it participates in stress defense by stabilizing thylakoid membrane structure and synergistically enhancing SOD and POD activities to scavenge reactive oxygen species—consistent with the cross-regulatory network of oxidative phosphorylation and plant hormone signal transduction pathways observed in multi-omics data. From the root–shoot co-regulation perspective, ABA may act as the key mediating signal: where drought stress significantly induced differential expression of ABA synthesis-related DEGs in roots and ABA signal transduction-related DEPs in leaves. Moreover, PPI network analysis showed that these ABA-related DEPs interact with the core hub gene *SHD*, suggesting that ABA may transmit root-derived stress signals to leaves via the vascular system, and then SHD mediates the transcriptional reprogramming of terpenoid biosynthesis-related DEGs (Table S5) to drive the biphasic response of L-borneol. Under drought stress, roots first perceive stress, synthesize ABA, and then either transport it to leaves via the vascular system or trigger transcriptional reprogramming of terpenoid biosynthesis-related genes to drive L-borneol’s biphasic response. The initial response at 8 days and peak content at 12 days of stress likely represent the spatiotemporal manifestation of continuous root signal input and synergistic activation of leaf metabolic pathways. While further validation is needed, this temporal coupling provides clues for dissecting *B. balsamifera*’s holistic drought response mechanism.

Drought stress induced a biphasic pattern of malondialdehyde (MDA) accumulation in *B. balsamifera* roots, with levels first increasing and then decreasing. This phenomenon reflects that excessive ROS accumulation drives membrane lipid peroxidation [48]. Plants employed two coordinated stress-response strategies: antioxidant defense and osmotic regulation [49]. In the antioxidant response, SOD and POD activities were significantly enhanced to scavenge ROS [50], while CAT activity exhibited a dynamic trend of initial increase followed by a decrease, consistent with CAT regulation patterns in oak seedlings under varying stress durations [51]. Regarding osmotic adjusters, PRO content reached 156.50 μg·g^−1^ at the end of treatment, potentially mitigating drought damage by stabilizing protein structures and maintaining cellular osmotic balance [52]. Correlation analysis revealed a significant positive correlation between MDA levels and antioxidant enzyme activities, indicating a close link between membrane lipid peroxidation and the antioxidant system. Coordinated changes in PRO and antioxidant enzyme activities further validated that *B. balsamifera* adapts to drought stress via ROS scavenging and osmotic regulation mechanisms.

Transcriptome analysis revealed that drought stress induced 9917 DEGs in *B. balsamifera*, with significant enrichment in phenylpropanoid biosynthesis, plant hormone signal transduction, and the MAPK signaling pathway. Among these, differential expression of phenylpropanoid pathway genes and LIG accumulation coordinately enhanced cell wall rigidity, consistent with mechanisms reported in *Malus domestica* (*MdMTA*-mediated lignin synthesis) [53] and *Maize* (*ZmNST3*-regulated cell wall biosynthesis gene expression) [54]. Importantly, integrated transcriptome–proteome analysis identified 12 DEG-DEP pairs in the phenylpropanoid biosynthesis pathway (e.g., cinnamyl alcohol dehydrogenase gene and its corresponding protein), which showed synchronized upregulation under drought stress. PPI network analysis further showed that these DEPs interact with the core hub gene *SHD*, suggesting that *SHD* may regulate cell wall structural remodeling by mediating the transcriptional and post-transcriptional expression of phenylpropanoid pathway genes/proteins. These findings indicate that cell wall structural remodeling represents a critical drought tolerance strategy in *B. balsamifera*. Proteomic analysis identified 736 DEPs significantly enriched in ATP-dependent chromatin remodeling, ABC transporters, and oxidative phosphorylation pathways.

Integrated transcriptome and proteome analysis identified 119 DEGs. PPI network analysis revealed 38 key hub genes, which may play core regulatory roles in drought response. Among these, TRINITY_DN9061_c0_g1 (*SHD*) and TRINITY_DN5849_c0_g1 (*CRT*) were the core hub genes screened from 63 DEGs-DEPs (differentially expressed genes–differentially expressed proteins) involved in 5 drought-related pathways: photosynthesis, endoplasmic reticulum protein processing, phagosome, photosynthetic carbon fixation, and plant–pathogen interaction. Studies show that *SHD* is involved in abiotic stress response pathways [55], while *CRT* regulates calcium signaling and stress responses [56]. Overexpression of *TaCRT* in *Triticum aestivum* has been shown to enhance drought tolerance [57]. Under drought stress, *SHD* is speculated to mediate stress signaling, linking photosynthetic carbon fixation and plant–pathogen interaction pathways, and signaling downstream modules for antioxidant enzyme and osmolyte synthesis to activate stress responses. *CRT* is thought to stabilize the photosynthetic apparatus function via the endoplasmic reticulum protein processing pathway by maintaining cellular calcium homeostasis. Specifically, *CRT* interacts with photosynthesis-related DEPs (e.g., *PsbA*, *PsaK*) in the PPI network, suggesting that it may regulate the folding and stability of these photosynthetic proteins at the post-transcriptional level, thereby mitigating drought-induced damage to the photosynthetic apparatus. These hypotheses will be validated using gene knockout or overexpression in model systems to clarify the molecular functions.

Changes in photosynthetic parameters of *B. balsamifera* indicate that damage to the photosynthetic apparatus represents a critical factor in its drought stress response. Leveraging this insight, the study focused on analyzing DEG-DEP pairs within the photosynthetic metabolic pathway, specifically examining synergistic regulatory patterns of core components such as Photosystem I/II, the cytochrome b_6_/f complex, photosynthetic electron transport chain, and ATP synthase.

Downregulation of D1 and D2 protein genes (*psbA*, *psbD*, etc.) in PSII was observed, consistent with findings in barley [58]. This downregulation is hypothesized to hinder oxygen evolution and electron transfer by weakening the stability of the water-splitting complex. Similarly, reduced expression of PSI subunit genes (*PsaA*, *PsaD*, *PsaE*, etc.) may induce oxidation of the PSI chlorophyll reaction center (P700), thereby decreasing the quantum efficiency of both PSII and PSI [59]. Genes encoding the cytochrome b6f complex (*PetA*, *PetB*, *PetC*) were significantly downregulated. As a core hub for electron transfer between PSII and PSI, this complex may mitigate excessive reduction in the PSI electron acceptor by slowing the plastid quinone (PQ) redox cycle [60]. The D1 protein, a critical component of the PSII reaction center linking plastid quinones QA and QB, showed *psbA*-mediated downregulation directly leading to a marked decline in photosynthetic electron transport chain (PETC) efficiency between QA and QB [61]. Collectively, these results indicate that inhibition of key photosynthetic apparatus components underlies reduced photosynthetic efficiency under drought stress. The candidate genes including *psbA*, *SHD* and *CRT* were screened based solely on differential expression and network analysis. Alterations in gene expression cannot fully confirm biological functions, as numerous drought-responsive genes are only passively induced and do not directly regulate drought tolerance. This study only conducted RT-qPCR validation of differential genes, lacking in-depth functional verification (e.g., virus-induced gene silencing, transgenic overexpression) of core candidate genes (*psbA*, *SHD*, *CRT*). Future studies will elucidate regulatory mechanisms of photosynthetic components and validate stress-resistant functions of key candidate genes and L-Borneol.

Furthermore, this study mainly focuses on the drought-responsive molecular mechanism of *B. balsamifera* roots. As key tissues for stress perception and signal transduction, leaves and stems can also independently initiate osmotic stress responses, whereas parallel multi-omics analysis of aboveground organs was absent in the present work, leaving organ-specific response patterns and inter-tissue signal coordination unclear. In addition, the damage of photosynthetic apparatus was inferred merely via differential expression of related genes and proteins, without direct structural and photochemical evidence such as chloroplast ultrastructure and chlorophyll fluorescence parameters. These deficiencies limit the systematic interpretation of the whole-plant drought adaptation strategy of *B. balsamifera*. Accordingly, future research will supplement multi-tissue omics analysis combined with photosynthetic physiological and ultrastructural detection, to clarify organ-specific drought response characteristics and further improve the regulatory network underlying drought tolerance.

## 5. Conclusions

Integrated physiological, biochemical, transcriptomic, and proteomic analyses were employed to dissect the drought response mechanisms in *B. balsamifera* roots. Under drought stress, leaf Pn, Tr, and Gs decreased significantly, while Ci increased, accompanied by coordinated downregulation of key Photosystem II genes (*psbA*), Photosystem I subunit genes, and cytochrome b6/f complex genes, confirming that photosynthetic apparatus damage drives non-stomatal limitations. L-Borneol content in leaves peaked at 12 days of stress, contributing to stress defense. Root MDA accumulation activated antioxidant enzymes (SOD, POD, CAT) to scavenge ROS, while osmotic regulators (PRO, SS, SP) maintained cellular homeostasis. Transcriptomic and proteomic profiling identified 9917 DEGs and 736 DEPs, significantly enriched in photosynthesis, plant hormone signal transduction, and phenylpropanoid biosynthesis pathways. Phenylpropanoid pathway genes and LIG accumulation likely synergistically enhance cell wall rigidity, with SHD and CRT acting as key nodes mediating signal transduction and calcium homeostasis. Downregulation of photosynthetic core components and electron transport chain DEG-DEP pairs impeded electron flow.

**Figure 9:**
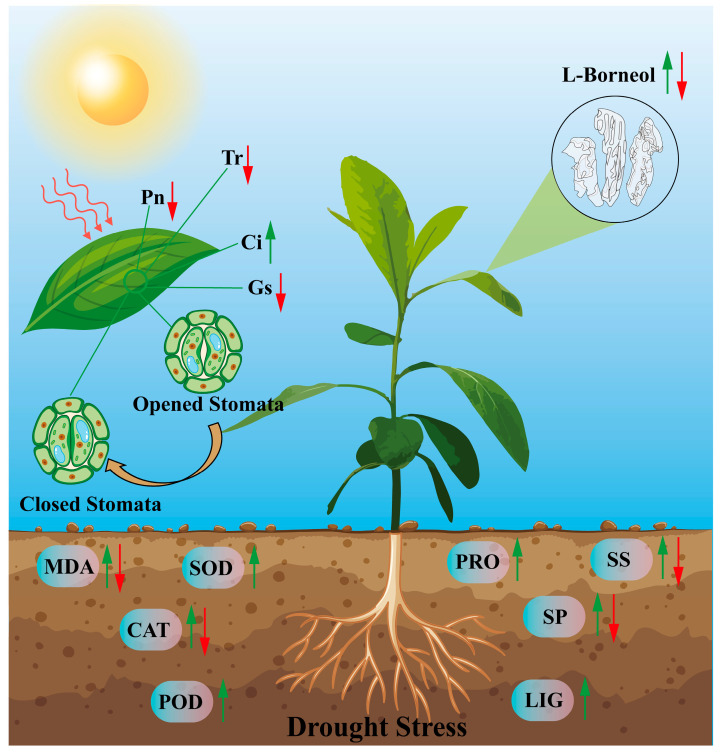
Network of drought mechanisms in *B. balsamifera.* Hypothesize the stomatal transition from opening to closure in leaves. The green upward arrow indicates that the activity or content of the substance increases with time; the red downward arrow indicates that the activity or content of the substance decreases with time; both upward and downward arrows indicate that the activity or content of the substance increases first and then decreases with time.

These findings reveal that *B. balsamifera* employs ROS scavenging, osmotic regulation, cell wall remodeling, and photosynthetic adjustment to confer drought tolerance (Figure 9), providing candidate genes (*psbA*, *SHD*, *CRT*,etc.) for drought-tolerant crop breeding. A limitation of this study is that while key pathways and genes were identified via multi-omics analyses, functional validation of these target genes has not been performed. Future work will verify the functions of these key genes via transgenic approaches, conduct field experiments, and evaluate the genes’ application value by integrating agronomic traits to support the breeding of drought-tolerant *B. balsamifera* varieties.

## Figures and Tables

**Table 1:** The mass fraction of L-Borneol in *B. balsamifera* leaves ω/(mg·g^−1^).

Processing Time (Day)	L-Borneol/(mg·g^−1^)		
	Control	Drought	Rewater
0	(0.066 ± 0.002) a	(0.078 ± 0.002) a	
5	(0.088 ± 0.001) a	(0.128 ± 0.001) a	
8	(0.059 ± 0.004) b	(0.186 ± 0.013) a	(0.166 ± 0.013) a
10	(0.066 ± 0.008) c	(0.752 ± 0.022) a	(0.081 ± 0.001) b
12	(0.107 ± 0.002) b	(0.969 ± 0.005) a	(0.106 ± 0.005) b
14	(0.089 ± 0.007) c	(0.431 ± 0.002) a	(0.152 ± 0.002) b
16	(0.065 ± 0.001) c	(0.223 ± 0.005) a	(0.157 ± 0.006) b

**Table note:** Different lowercase letters at the same processing time indicate significant differences among different treatment groups (*p* < 0.05), and the same lowercase letters mean no significant differences among different treatment groups (*p* > 0.05).

## Data Availability

All datasets generated and analyzed in this study are included in the present manuscript and its supplementary files. Raw RNA sequencing data have been deposited in the NCBI public repository, accessible via the link: https://www.ncbi.nlm.nih.gov/sra/PRJNA1252783 (accessed on 6 December 2025). The proteomic datasets can be retrieved from the PRIDE database with the project accession PXD063289 and access token nRQozWNwyHkU. Reviewers are also able to log into the PRIDE platform to access the data using the dedicated account: Username: reviewer_pxd063289@ebi.ac.uk, Password: NR1aP2oJ6dBy.

## Supplementary Materials

The following supporting information can be downloaded at: https://www.mdpi.com/article/10.3390/biology15110861/s1. Table S1. Genes and corresponding primers used in the qPCR test. Table S2. Quality control statistics of sequencing data. Table S3. Comparison of sequencing results with assembly results. Table S4. Top 10 genes in PPI network. Table S5. Terpenoid biosynthesis pathway-related genes.

## Abbreviations

The following abbreviations are used in this manuscript:

ROS	Reactive oxygen species
ABA	Abscisic acid
Pn	Photosynthetic rate
Gs	Stomatal conductance
Tr	Transpiration rate
Ci	Intercellular CO_2_ concentration
MDA	Malondialdehyde
SOD	Superoxide
CAT	Catalase
POD	Peroxidase
LIG	Lignin
SS	Soluble sugar
SP	Soluble protein
PRO	Proline
GO	Gene Ontology
KEGG	Kyoto Encyclopedia of Genes and Genomes
RT-qPCR	Quantitative reverse transcription polymerase chain reaction
PSI	Photosystem I
PSII	Photosystem II

## References

[1] Liu J., Liao J., Li C. (2022). Bottom-up effects of drought on the growth and development of potato, *Leptinotarsa decemlineata* Say and *Arma chinensis* Fallou. Pest Manag. Sci..

[2] Yang L., Zhao Y., Zhang Q., Cheng L., Han M., Ren Y., Yang L. (2019). Effects of drought-re-watering-drought on the photosynthesis physiology and secondary metabolite production of *Bupleurum chinense* DC. Plant Cell Rep..

[3] Wang H., Wang Y., Kang C., Wang S., Zhang Y., Yang G., Zhou L., Xiang Z., Huang L., Liu D. (2022). Drought stress modifies the community structure of root-associated microbes that improve *Atractylodes lancea* growth and medicinal compound accumulation. Front. Plant Sci..

[4] Di Sario L., Boeri P., Matus J.T., Pizzio G.A. (2025). Plant Biostimulants to Enhance Abiotic Stress Resilience in Crops. Int. J. Mol. Sci..

[5] Xie Z., Jin L., Sun Y., Zhan C., Tang S., Qin T., Liu N., Huang J. (2024). OsNAC120 balances plant growth and drought tolerance by integrating GA and ABA signaling in rice. Plant Commun..

[6] Wilmowicz E., Kućko A., Alché J.D., Czeszewska-Rosiak G., Florkiewicz A.B., Kapusta M., Karwaszewski J. (2022). Remodeling of Cell Wall Components in Root Nodules and Flower Abscission Zone under Drought in Yellow Lupine. Int. J. Mol. Sci..

[7] Wang D., Zhang X., Cao Y., Batool A., Xu Y., Qiao Y., Li Y., Wang H., Lin X., Bie X. (2024). TabHLH27 orchestrates root growth and drought tolerance to enhance water use efficiency in wheat. J. Integr. Plant Biol..

[8] Ren Y., Yang G., Gao G.L., Ding G.D. (2026). Dynamic physiological response of Mongolian pine ectomycorrhizal seedlings to drought and re-watering. Front. Plant Sci..

[9] Yan M., Chai M., Li L., Dong Z., Jin H., Tan M., Ye Z., Yu S., Feng Z. (2024). Calcium-Dependent Protein Kinase GhCDPK16 Exerts a Positive Regulatory Role in Enhancing Drought Tolerance in Cotton. Int. J. Mol. Sci..

[10] Li J., Wu Q., Cheng J., Zhu J., Su P., Wu J., Fan X., Li G. (2025). Mechanism of Exogenous Dopamine Regulating Shine Muscat Grape in Response to Low-Temperature Stress. Plants.

[11] Pilon C., Snider J.L., Sobolev V., Chastain D.R., Sorensen R.B., Meeks C.D., Massa A.N., Walk T., Singh B., Earl H.J. (2018). Assessing stomatal and non-stomatal limitations to carbon assimilation under progressive drought in peanut (*Arachis hypogaea* L.). J. Plant Physiol..

[12] Panda S.K., Gupta D., Patel M., Vyver C.V., Koyama H. (2024). Functionality of Reactive Oxygen Species (ROS) in Plants: Toxicity and Control in Poaceae Crops Exposed to Abiotic Stress. Plants.

[13] Araújo E.D., Soares L., Lima G.S., Nunes K.G., Costa D.S., Souza A.R., Souza N.P., Borborema L.D.A., Arruda T.F.L., da Silva F.A. (2026). Effects of a Hydrogel Polymer on the Physiology and Antioxidant Activity of Naturally Colored Cotton Cultivars Under Water Deficit. Plants.

[14] Rehman S., Rashid A., Manzoor M.A., Li L., Sun W., Riaz M.W., Li D., Zhuge Q. (2021). Genome-Wide Evolution and Comparative Analysis of Superoxide Dismutase Gene Family in Cucurbitaceae and Expression Analysis of *Lagenaria siceraria* Under Multiple Abiotic Stresses. Front. Genet..

[15] Lü X.P., Gao H.J., Zhang L., Wang Y.P., Shao K.Z., Zhao Q., Zhang J.L. (2019). Dynamic responses of *Haloxylon ammodendron* to various degrees of simulated drought stress. Plant Physiol. Biochem..

[16] Ahanger M.A., Bhat J.A., Siddiqui M.H., Rinklebe J., Ahmad P. (2020). Integration of silicon and secondary metabolites in plants: A significant association in stress tolerance. J. Exp. Bot..

[17] Feng W., Liu Y., Cao Y., Zhao Y., Zhang H., Sun F., Yang Q., Li W., Lu Y., Zhang X. (2022). Maize ZmBES1/BZR1-3 and -9 Transcription Factors Negatively Regulate Drought Tolerance in Transgenic *Arabidopsis*. Int. J. Mol. Sci..

[18] Kong L., Chen P., Chang C. (2023). Drought Resistance and Ginsenosides Biosynthesis in Response to Abscisic Acid in *Panax ginseng* C. A. Meyer. Int. J. Mol. Sci..

[19] Wang T., Wei Q., Wang Z., Liu W., Zhao X., Ma C., Gao J., Xu Y., Hong B. (2022). CmNF-YB8 affects drought resistance in chrysanthemum by altering stomatal status and leaf cuticle thickness. J. Integr. Plant Biol..

[20] Zhang Y., Zhang H., Zhang Y., Wang D., Meng X., Chen J. (2024). Utilizing physiologies, transcriptomics, and metabolomics to unravel key genes and metabolites of *Salvia miltiorrhiza* Bge. seedlings in response to drought stress. Front. Plant Sci..

[21] Guan L., Lin N., Wan L., Yu F., Chen X., Xie X., Yuan C., Soaud S.A., Abd Elhamid M.A., Heakel R.M.Y. (2024). Transcriptome analysis revealed the role of moderate exogenous methyl jasmonate treatments in enhancing the metabolic pathway of L-borneol in the *Blumea balsamifera*. Front. Plant Sci..

[22] Yuan Y., Tang W.J., Cao J.Y., Zhong K., Mo Z.J., Zhou Y., Pang Y.X. (2024). Integrated physiological and transcriptomic analysis uncovers the mechanism of moderate nitrogen application on promoting the growth and (-)-borneol accumulation of *Blumea balsamifera*. Front. Plant Sci..

[23] Guan L., Yang Y., Jiang P., Mou Q., Gou Y., Zhu X., Xu Y.W., Wang R. (2022). Potential distribution of *Blumea balsamifera* in China using MaxEnt and the ex situ conservation based on its effective components and fresh leaf yield. Environ. Sci. Pollut. Res..

[24] Terletskaya N.V., Shcherban A.B., Nesterov M.A., Perfil’ev R.N., Salina E.A., Altayeva N.A., Blavachinskaya I.V. (2020). Drought Stress Tolerance and Photosynthetic Activity of Alloplasmic Lines *T. dicoccum* x *T. aestivum*. Int. J. Mol. Sci..

[25] Li Y., Hu W., Zou J., He J., Zhu H., Zhao W., Wang Y., Chen B., Meng Y., Wang S. (2023). Effects of soil drought on cottonseed kernel carbohydrate metabolism and kernel biomass accumulation. Plant Physiol. Biochem..

[26] Li J., Han G., Sun C., Sui N. (2019). Research advances of MYB transcription factors in plant stress resistance and breeding. Plant Signal. Behav..

[27] Wang H., Ni D., Shen J., Deng S., Xuan H., Wang C., Xu J., Zhou L., Guo N., Zhao J. (2022). Genome-Wide Identification of the AP2/ERF Gene Family and Functional Analysis of GmAP2/ERF144 for Drought Tolerance in Soybean. Front. Plant Sci..

[28] Yu F.L., Zhao N., Wu Z.S., Huang M., Wang D., Zhang Y.B., Hu X., Chen X.L., Huang L.Q., Pang Y.X. (2017). NIR Rapid Assessments of *Blumea balsamifera* (Ai-na-xiang) in China. Molecules.

[29] Yao Y., Nan L., Wang K., Xia J., Ma B., Cheng J. (2024). Integrative leaf anatomy structure, physiology, and metabolome analyses revealed the response to drought stress in sainfoin at the seedling stage. Phytochem. Anal..

[30] Lu R., Zhang T., Wu D., He Z., Jiang L., Zhou M., Cheng Y. (2018). Production of functional human CuZn-SOD and EC-SOD in bitransgenic cloned goat milk. Transgenic Res..

[31] Wang S., Gu H., Chen S., Li Y., Shen J., Wang Y., Ding Z. (2023). Proteomics and phosphoproteomics reveal the different drought-responsive mechanisms of priming with (Z)-3-hexenyl acetate in two tea cultivars. J. Proteom..

[32] Qiao C., Wang X., Gao Y., Li J., Zhao J., Luo H., Zhang S., Huo D., Hou C. (2024). A novel colorimetric and fluorometric dual-signal identification of organics and Baijiu based on nanozymes with peroxidase-like activity. Food Chem..

[33] Li D., Yang J., Pak S., Zeng M., Sun J., Yu S., He Y., Li C. (2022). PuC3H35 confers drought tolerance by enhancing lignin and proanthocyanidin biosynthesis in the roots of *Populus ussuriensis*. New Phytol..

[34] Zhang Y., Cheng W., Di H., Yang S., Tian Y., Tong Y., Huang H., Escalona V.H., Tang Y., Li H. (2024). Variation in Nutritional Components and Antioxidant Capacity of Different Cultivars and Organs of *Basella alba*. Plants.

[35] Okumoto K., Tamura S., Fujiki Y. (2017). Blue Native PAGE: Applications to Study Peroxisome Biogenesis. Peroxisomes.

[36] Magné C., Larher F. (1992). High sugar content of extracts interferes with colorimetric determination of amino acids and free proline. Anal. Biochem..

[37] Chen S., Zhou Y., Chen Y., Gu J. (2018). fastp: An ultra-fast all-in-one FASTQ preprocessor. Bioinformatics.

[38] Li Y., Zhao M., Cai K., Liu L., Han R., Pei X., Zhang L., Zhao X. (2023). Phytohormone biosynthesis and transcriptional analyses provide insight into the main growth stage of male and female cones *Pinus koraiensis*. Front. Plant Sci..

[39] Deng S., Ma J., Zhang L., Chen F., Sang Z., Jia Z., Ma L. (2019). De novo transcriptome sequencing and gene expression profiling of *Magnolia wufengensis* in response to cold stress. BMC Plant Biol..

[40] Gu X., Wang S., Zhang W., Li C., Guo L., Wang Z., Li H., Zhang H., Zhou Y., Liang W. (2023). Probing long COVID through a proteomic lens: A comprehensive two-year longitudinal cohort study of hospitalised survivors. EBioMedicine.

[41] Meng C., Yang M., Wang Y., Chen C., Sui N., Meng Q., Zhuang K., Lv W. (2020). SlWHY2 interacts with SlRECA2 to maintain mitochondrial function under drought stress in tomato. Plant Sci..

[42] Malik A.I., Storey J.M., Storey K.B. (2023). Regulation of the unfolded protein response during dehydration stress in African clawed frogs, *Xenopus laevis*. Cell Stress Chaperones.

[43] Li L., Yi H. (2022). Enhancement of drought tolerance in Arabidopsis plants induced by sulfur dioxide. Ecotoxicology.

[44] Wang R., Qin X., Pan H., Li D., Xiao X., Jin Y., Wang Y., Liang H. (2025). Assessing the effects of drought stress on photosynthetic performance and physiological resistance in camphor seedling leaves. PLoS ONE.

[45] Wang J., Wan X., Liu Q., Zhang Y., Tian B., Chen C., Gu R., Wang B., Chen J., Chen L. (2025). Physiological and molecular mechanism analysis of *Cyclocodon lancifolius* seedlings in response to varying degrees of drought stress. BMC Plant Biol..

[46] Wei X., Cao K., Lu X., Lu R., Li L., Huang R., He Y., Chen J., Xiao J. (2026). Integrative physiological and transcriptomic analysis reveals drought response mechanisms in *Abrus mollis* Hance. BMC Plant Biol..

[47] Huang C., Liao J., Huang W., Qin N. (2022). Salicylic Acid Protects Sweet Potato Seedlings from Drought Stress by Mediating Abscisic Acid-Related Gene Expression and Enhancing the Antioxidant Defense System. Int. J. Mol. Sci..

[48] Liu S., Zhang F., Feng H., Wang X., Wang Q., Lai X., Yan L. (2025). StTCTP Positively Regulates StSN2 to Enhance Drought Stress Tolerance in Potato by Scavenging Reactive Oxygen Species. Int. J. Mol. Sci..

[49] Khan R., Ma X., Zhang J., Wu X., Iqbal A., Wu Y., Zhou L., Wang S. (2021). Circular drought-hardening confers drought tolerance via modulation of the antioxidant defense system, osmoregulation, and gene expression in tobacco. Physiol. Plant..

[50] Farooq M.A., Islam F., Ayyaz A., Chen W., Noor Y., Hu W., Hannan F., Zhou W. (2022). Mitigation effects of exogenous melatonin-selenium nanoparticles on arsenic-induced stress in *Brassica napus*. Environ. Pollut..

[51] Yan X.F., Deng X.J., Wang J., Zhou L.B., Zhang J.F., Luo Y.H. (2020). Effects of seed size and drought stress on the growth and physiological characteristics of *Quercus wutaishanica* seedlings. J. Appl. Ecol..

[52] Wang H., Li N., Li H., Zhang S., Zhang X., Yan X., Wang Z., Yang Y., Zhang S. (2023). Overexpression of NtGCN2 improves drought tolerance in tobacco by regulating proline accumulation, ROS scavenging ability, and stomatal closure. Plant Physiol. Biochem..

[53] Hou N., Li C., He J., Liu Y., Yu S., Malnoy M., Mobeen Tahir M., Xu L., Ma F., Guan Q. (2022). MdMTA-mediated m(6) A modification enhances drought tolerance by promoting mRNA stability and translation efficiency of genes involved in lignin deposition and oxidative stress. New Phytol..

[54] Ren Z., Zhang D., Cao L., Zhang W., Zheng H., Liu Z., Han S., Dong Y., Zhu F., Liu H. (2020). Functions and regulatory framework of ZmNST3 in maize under lodging and drought stress. Plant Cell Environ..

[55] Ojosnegros S., Alvarez J.M., Grossmann J., Gagliardini V., Quintanilla L.G., Grossniklaus U., Fernández H. (2022). The Shared Proteome of the Apomictic Fern *Dryopteris affinis* ssp. *affinis* and Its Sexual Relative *Dryopteris oreades*. Int. J. Mol. Sci..

[56] Muhammad T., Yang T., Wang B., Yang H., Tuerdiyusufu D., Wang J., Yu Q. (2024). Comprehensive genomic characterization and expression analysis of calreticulin gene family in tomato. Front. Plant Sci..

[57] Wang Y., Yang P., Li J., Li J., Zhu K., Pei M., Li J., Du H. (2025). Identification of the CaCRT gene family and function of CaCRT1 under low-temperature stress in pepper (*Capsicum annuum* L.). Sci. Rep..

[58] Harb A., Simpson C., Guo W., Govindan G., Kakani V.G., Sunkar R. (2020). The Effect of Drought on Transcriptome and Hormonal Profiles in Barley Genotypes With Contrasting Drought Tolerance. Front. Plant Sci..

[59] Zhu Z., Zhang H., Tian H., Chai G., Muhammad R., Wang Q., Liang B., Wu X. (2025). Comprehensive analysis of the effects on photosynthesis and energy balance in tomato leaves under magnesium deficiency. Plant Physiol. Biochem..

[60] Miao Y., Gao X., Li B., Wang W., Bai L. (2022). Low red to far-red light ratio promotes salt tolerance by improving leaf photosynthetic capacity in cucumber. Front. Plant Sci..

[61] Yan K., Mei H., Dong X., Zhou S., Cui J., Sun Y. (2022). Dissecting photosynthetic electron transport and photosystems performance in Jerusalem artichoke (*Helianthus tuberosus* L.) under salt stress. Front. Plant Sci..

